# The burden of hepatitis E virus infection among Ghanaian pregnant women

**DOI:** 10.3389/fpubh.2024.1507488

**Published:** 2025-01-07

**Authors:** Husein Bagulo, Ayodele O. Majekodunmi, Susan C. Welburn, Langbong Bimi

**Affiliations:** ^1^Zhejiang University – University of Edinburgh Institute, Haining, Zhejiang, China; ^2^School of Biomedical Sciences, Edinburgh Medical School, College of Medicine and Veterinary Medicine, The University of Edinburgh, Edinburgh, United Kingdom; ^3^Food and Agriculture Organisation of the United Nations, Garki, Abuja, Nigeria; ^4^Department of Animal Biology and Conservation Science, College of Basic and Applied Sciences, University of Ghana, Legon, Accra, Ghana

**Keywords:** hepatitis E virus (HEV), seroprevalence, risk facors, pregnant women, poor pregnancy outcomes

## Abstract

**Introduction:**

Hepatitis E virus (HEV) infection poses a significant burden on pregnant women, with associated negative outcomes. Although well-described in many developed countries, the epidemiology of the disease and its impact on maternal and fetal health in Ghana is not fully understood.

**Materials and methods:**

A cross-sectional survey was conducted in the antenatal clinics of 10 district hospitals in five regions of Ghana. The study involved 1,000 pregnant women attending antenatal care. Serological and virological assays were employed to determine HEV seroprevalence and prevalence. Logistic regression analysis was carried out in univariate and multivariate models to assess risk factors associated with HEV infection.

**Results:**

HEV-Immunoglobulin G (IgG) seroprevalence of 8.3% was recorded among the pregnant women with 1% HEV-antigen prevalence. However, none were positive for HEV-IgM and HEV RNA. 19.8% of the pregnant women reported poor pregnancy outcomes in previous pregnancies. Age, educational attainment, and region were significant predictors of HEV IgG seropositivity in the univariate regression model, while age and region were the only significant predictors in a multivariate model. Also, the drinking water source and the toilet type accurately predicted HEV IgG seroprevalence in both univariate and multivariate models.

**Discussion:**

Pregnancy care must be significantly improved to reduce maternal and foetal morbidity and mortality.

## Introduction

Viral hepatitis is a global public health issue and poses a significant burden on lives, communities, and health systems (1). It is the cause of an estimated 1.4 million deaths every year worldwide through acute infections and other hepatitis-related conditions (1). Moreover, over 900 million individuals have experienced infections throughout their lifetime, with approximately 15–110 million currently experiencing recent or ongoing infections (2). HEV is a significant cause of death among people living with HIV (1). Among the viral hepatitis viruses, hepatitis E virus (HEV) infection tops acute infections in developing countries (3–5). Infections are, however, not confined to specific developing nations; they are also endemic in numerous high-income countries and are predominantly zoonotic (6). Although the disease is usually mild in healthy individuals, pregnant women, persons with underlying health conditions and altered immunological changes could experience fatal outcomes (7, 8). HEV has more serious consequences among pregnant women due to hormonal changes during pregnancy, and there is a risk of vertical transmission to the foetus (6, 9). Maternal and fetal morbidities and mortalities often accompany infections in pregnant women in fulminant cases (9–11).

Several seroprevalence studies worldwide have shown that HEV infection is high among pregnant women, accompanied by a risk of fulminant hepatitis with a mortality rate of between 30 and 100% (12). However, only two studies have investigated HEV seroprevalence among pregnant women in Ghana, although many studies report high seroprevalence among the general public and swine farmers. In both studies, significantly higher seroprevalences were recorded during the third trimester of pregnancy than in the first and second trimesters; 30.2% in the Greater Accra region (13) and 55.3% in the Central region (14). Also, a case report revealed among three pregnant women in Ghana with fulminant hepatitis E, 33.3% spontaneous abortion, 33.3% miscarriage, 33.3% icteric baby, and 66.7% maternal mortality (15).

While these studies indicate a high burden of infection in pregnant women in Ghana, more studies are required to understand the epidemiology and device prevention and control measures in this disease-vulnerable population. This becomes even more important as Ghana looks to improve its maternal and neonatal mortalities by 2050 as part of the SDG targets. HEV investigation in pregnant women and prevention should, therefore, be seriously considered as it is a major contributor to maternofetal mortality. This study aims to investigate HEV seroprevalence, risk factors for transmission, and poor pregnancy outcomes of HEV infection to determine the burden of the disease on pregnant women in Ghana.

## Materials and methods

### Study design and sample size determination

The sampling plan and survey methodology were developed along with an epidemiological survey, to determine the contribution of zoonotic and water, sanitation and hygiene-related transmission routes to the burden of hepatitis E in Ghana between October 2021 and December 2022. Briefly, the study included 16 communities in five regions in the southeastern and northern parts of Ghana. The regions included the Greater Accra Region, the Volta Region, the Eastern Region, the Central Region, and the Northern Region. These were areas where people commonly bred free-roaming pigs. Two districts were randomly selected from each region in the first stage, and two communities with the desired sanitation level were selected from each district in the second stage. The pregnant women were sampled from the antenatal clinics of district hospitals in the selected districts. These antenatal clinics are the common point of call and receive pregnant women across the districts. The sample size was determined using Cochran’s Formula (16).

$\mathrm{The Cochran formula}:n_{o}=\frac{Z^{2}pq}{e^{2}}$

where

*n_o_*: estimated sample size, *Z*: value from a typical normal distribution that matches the necessary level of confidence (*Z* = 1.96 for 95% CI), *p*: the percentage of the population that possesses the questioned characteristic, *q*: the proportion of the population without the attribute in question (*q* = 1 − *p*), and *e*: desired degree of accuracy (half desired CI width = 0.05%).

A HEV seroprevalence value of 12.2% (14) determined among pregnant women in the Central Region of Ghana was used. Assuming a normally distributed population with no clustering among the pregnant women, a sample size of 165 was estimated from the above formula. However, the sample size was adjusted upwards by 20% (17) to cater for attrition and non-response by respondents, giving a sample size of 198. The sample size was finally, rounded up to 200. This sample size was used as the sample size for each region. Since two district hospitals were selected from each region, the sample size was divided equally. Thus, 100 pregnant women made up the final sample size for each district hospital.

### Sampling technique and data collection

Consecutive pregnant women attending antenatal care were recruited. The purpose and nature of the study and information regarding voluntary participation, benefits, risks, compensation, confidentiality, cost, and time involved were explained to all respondents in the language they best understood, and questions were addressed. Voluntary informed consent was acquired from all respondents before participation in the study. A sample of blood was collected from each pregnant woman, and information on demographics: age, education level, occupation; water, sanitation, and hygiene (WASH)—drinking water source and toilet type; contact with pig and pork products—nature of pig or pork contact; pregnancy history, and neonatal outcomes, as well as obstetric and other relevant parameters: number of past pregnancies, outcomes, and children, and gestation and pregnancy complications was gathered using a structured questionnaire. The questionnaires underwent pre-testing with 20 pregnant women at the University Hospital, University of Ghana, Legon, Accra, Ghana.

### Serological and virological tests

Aliquots of sera of the blood samples of all the pregnant women were tested serologically for anti-HEV IgG and IgM antibodies and positive samples were tested for HEV antigen by ELISA. All antigen-positive samples were tested for HEV RNA using a real-time PCR assay. The ELISAs were acquired from Wantai Biological Pharmacy Enterprise Co., Beijing, China. The Wantai HEV IgG ELISA has a sensitivity of 99.08% and a specificity of 99.90%, while the IgM ELISA have 97.10 and 98.40% (18).

### HEV IgM/IgG ELISA

Using an indirect ELISA method, the WANTAI HEV-IgM/IgG ELISAs employ a two-step incubation procedure to detect IgM/IgG-class antibodies to HEV (anti-HEV). Polystyrene microwell strips were pre-coated with recombinant HEV ORF2 antigens specific to anti-HEV immunoglobulin M/G. Sera were added to the microwells and incubated for 60 min at 37°C. HEV-specific antibodies, if present in the sera, will bind to the immobilized HEV antigens. Excess and unbound serum proteins were removed by washing the wells, and an anti-human IgG/IgM antibody conjugated to horseradish peroxidase (HRP-Conjugate) was added and incubated for 30 min at 37°C. All previously formed antigen–antibody complexes will bind to these HRP-conjugated antibodies during the second incubation step, after which the unbound HRP-conjugates are washed away. Tetramethyl benzidine (TMB) and urea peroxide-containing chromogen solutions were added to the wells. In the presence of the antigen–antibody-anti-IgG/IgM-HRP immunocomplexes, the bound HRP conjugates hydrolyse the colorless chromogens to produce blue-colored products. Sulphur acid was added to stop the reactions, turning the blue color yellow. The color intensity was measured at a wavelength of 450 nm and was proportionate to the amount of antibodies captured in the wells, and, thus, the amount in the serum samples. Wells with specimens that tested negative for HEV-IgG/IgM stay colorless. The cut-off (CO) value was computed using the formula CO = Nc + 0.26 for IgM and C.O = Nc + 0.16 for IgG, where Nc is the mean absorbance of three negative controls. The results were interpreted as positive and negative.

The HEV antigen assay follows a similar protocol.

The antigen assay can detect HEV antigens in serum, plasma and stool samples. The identification of HEV antigens serves as an indicator of the virus’s existence and exhibits a more robust association with ongoing HEV infection (19). The Wantai Antigen ELISA (Wantai Biological Pharmacy Enterprise Co., Beijing, China) was validated by testing 1,494 clinical samples, including those from patients with hepatitis A, B, C, and Syphilis, as well as samples from patients with chronic hepatitis whose infection type were unknown. The assay was also used to test 251 samples that were collected from three different centers in China, and the results were verified by PCR. The assay has a sensitivity of 99.08% and a specificity of 99.90% (18).

### Detection of HEV RNA

Following diligently the guidelines provided by the manufacturer, viral RNA was extracted from 140 μL of serum using QIAamp viral RNA microkit from Qiagen, Hilden, USA. Then, HEV RNA was eluted using 50 μL of the elution buffer provided with the kit for use in the PCR reaction. Total RNA was subjected to a real-time RT-PCR with a Real Star^®^ HEV RT-PCR Kit 2.0 (Altona Diagnostics, GmbH, Hamburg, Germany), using a reverse primer (5′-AGG GGT TGG TTG GAT GAA-3′) and forward a primer (5′-GGT GGT TTC TGG GGTGAC-3′). A 5′ reporter dye (FAM) and a 3′-quencher dye (JOE) - (5′-FAM-TGA TTC TCA GCC CTT CGC-JOE-3′) was used as the probe.

RNA transcription was performed at 50°C for 30 min, followed by a single cycle of 95°C for 15 min for denaturation, 50 cycles of 94°C for 15 s for amplification, 56°C for 30 s, and 76°C for 30 s. The subsequent thermal cycling conditions for the PCR were as follows, 94°C for 15 min for denaturation, 50 cycles of 56°C for 30 s for annealing, and 76°C for 30 s for extension.

Known HEV RNA negative and positive samples were used as controls and internal controls for quality assurance.

### Ethical considerations

The Ghana Health Services Ethical Review Committee (GHS-ERC013/10/19) and the Ethics Committee of the College of Basic and Applied Sciences at the University of Ghana (ECBAS 003/19–20) both granted their clearance for the entire study. Written permission was sought from administrators of hospitals and in-charges of the antenatal clinics. All research respondents provided written informed consent. The study was conducted following the 2024 Declaration of Helsinki on Human Experimentation.

### Data analysis

Serological test results and respondents’ data from the structured questionnaires were imported into Microsoft Excel. The statistical analysis was performed using the Statistical Package for Social Sciences (SPSS) version 26.0 software (New York, United States). Demographic descriptive data were calculated, and the output was shown as percentages.

Categorical variables were compared using the Pearson chi-squared (*χ*^2^) test, also used to determine statistically significant differences in seroprevalence between the various groups. *p* < 0.05 was adopted as the threshold for significance.

Logistic regression analysis was carried out in univariate and multivariate models, and odds ratios (ORs) were used to determine risk factors for HEV infection, along with their corresponding 95% CIs. The chi-squared test of the likelihood ratio test (LRT), which measures the goodness of fit of the overall regression model was used to assess the contribution of each variable to the model (20). In majority of the multivariate regression models, the variables to be included are first assessed for significance in a univariate regression model. However, there are several reasons why non-significant variables in a univariate model may become significant in a multivariate model (21). Therefore, all variables used in the univariate model were entered in a single step in the final multivariate model (Table 1).

**Table 1:** Regions, districts, hospitals and number of pregnant women sampled.

Region	District/municipality	Hospital	*N*
Accra	Ada East	Ada East District Hospital	100
	Shai-Osudoku	Shai-Osudoku District Hospital	100
Volta	North Tongu	Catholic Hospital, Battor	100
	Keta	Keta Municipal Hospital	100
Central	Cape Coast	Ewim Poly Clinic	100
	Ajumako District	Ajumako District Hospital	100
Eastern	Nsawam Adoagyiri	Notre Dame Clinic	100
	Fanteakwa District	Begoro District Hospital	100
Northern	Sagnarigu	St Lucy Polyclinic	100
	Saboba	Assemblies of God Hospital	100

## Results

A total of 1,000 pregnant women accessing routine antenatal services were sampled from 10 district hospitals in five regions of Ghana, with 200 pregnant women from each region. A total of 178 were in their first trimester, 355 in their second, and 467 in their third. The majority (50.5%) of the pregnant women had primary and some secondary school education, 9.4% were tertiary school graduates, and 21.2% were senior high school leavers. 18.9% of the women in the study were not literate. Regarding employment, 4.1, 8.9, 57.1, and 11.9% were engaged as students, public servants, traders, and farmers, respectively, while 18.0% were unemployed. Their ages ranged from 13 to 60 years, with a mean age ± standard deviation (SD) of 28.1 ± 6.4 years. The demographic characteristics of the pregnant women and HEV seroprevalence are presented in Table 2.

**Table 2:** Sociodemographic characteristics of pregnant women.

Demographic variables	*N*	Percentage (mean of age)	HEV IgG seroprevalence	
			%	(95% CI)
Age group (years)				
<20	74	7.4% (17.6)	2.7	(0.74–9.33)
20–25	293	29.3% (22.4)	8.5	(5.85–12.29)
26–30	300	30.0% (28.0)	6.0	(3.83–9.28)
31–35	186	18.6% (32.9)	7.5	(4.54–12.24)
36–40	124	12.4% (37.8)	16.1	(10.69–23.60)
>40	23	2.3% (43.7)	17.4	(6.98–37.14)
Stage of pregnancy				
Fisrt trimester	178	17.8%	6.2	(3.49–10.73)
Second trimester	355	35.5%	8.5	(5.98–11.81)
Third trimester	467	46.7%	9.0	(6.72–11.93)
Educational level				
Basic education	505	50.5%	3.6	(2.27–5.56)
Senior high school education	212	21.2%	8.0	(5.07–12.47)
Tertiary education	94	9.4%	12.8	(7.46–21.00)
No formal education	189	18.9%	19.0	(14.09–25.24)
Employment				
Student	41	4.1%	2.4	(0.43–12.60)
Public servant	89	8.9%	12.4	(7.04–20.79)
Traders	571	57.1%	8.8	(6.71–11.36)
Farmer	119	11.9%	9.2	(5.24–15.80)
Unemployed	180	18.0	5.6	(3.05–9.92)
Region				
Greater Accra	200	20.0%	3.5	(1.71–7.05)
Central	200	20.0%	2.5	(1.07–5.72)
Volta Region	200	20.0%	1.5	(0.51–4.32)
Eastern	200	20.0%	6.0	(3.47–10.19)
Northern	200	20.0%	28.0	(22.24–34.59)
Total	1,000	–	8.3	(6.75–10.17)

**Figure 1:**
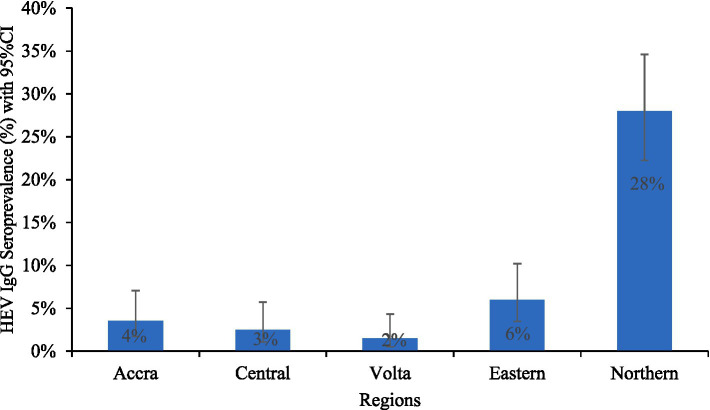
HEV IgG seroprevalence among pregnant women in regions.

The overall HEV IgG seroprevalence was 8.3%. Only 1.0% of the individuals tested positive for HEV antigen (HEV-Ag), while no pregnant woman tested positive for HEV-IgM or HEV RNA. Of those positive for HEV-Ag, 80% were from the Northern Region. HEV IgG seroprevalence did not differ significantly among the pregnant women regarding the stage of pregnancy or type of employment. Thus, there was no association between these parameters and HEV IgG seroprevalence. However, the HEV IgG seroprevalence was highest in the Northern Region (28.0%) and lowest in the Volta Region (1.5%). The seroprevalence was 3.5, 6.0, and 2.5% in the Greater Accra, the Eastern, and the Central regions, respectively (Figure 1). The HEV IgG seroprevalence was significantly higher in the Northern Region than in all the other regions, *χ*^2^ (4) = 130.415, *p* < 0.0001, and considerably higher in the Eastern Region than in the Volta Region, *χ*^2^ (1) = 5.596; *p* = 0.0180.

**Figure 2:**
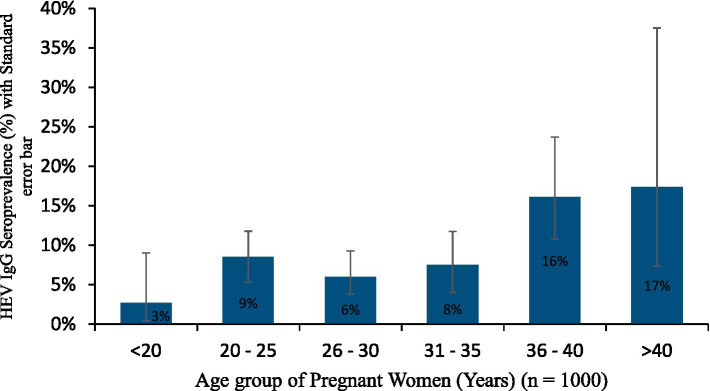
HEV IgG seroprevalence in the age group of pregnant women.

There was also, a significantly higher HEV IgG seroprevalence in those aged 36–40 years (16.1%) than in the <20 years (2.7%), 20–25 years (8.5%), 26–30 years (6.0%), and 31–35 years (7.5%), while it was significantly higher in those aged >40 years (17.4%) than in the <20 years (2.7%) and 26–30 years (6.0%) groups, *p* < 0.05. As shown in Table 2 and Figure 2, the seroprevalence increased with increasing age.

Moreover, the HEV IgG seroprevalences in pregnant women with senior high school education (8.0%), tertiary education (12.8%), and those with no formal education (19.0%) were significantly higher than in those with basic education, *χ*^2^ (3) = 46.049; *p* < 0.0001, whereas those with no formal education had a significantly higher seroprevalence than those with senior high school education, *χ*^2^ (1) = 10.570; *p* = 0.0011.

**Figure 3:**
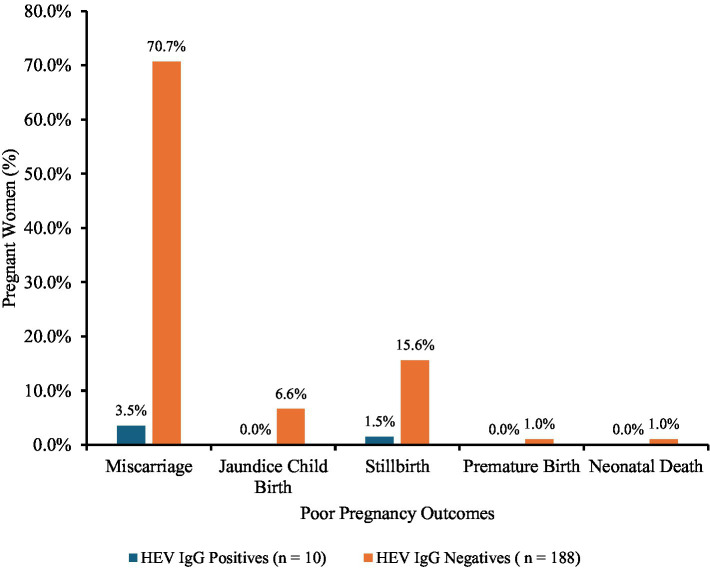
Poor pregnancy outcomes in pregnant women. Poor pregnancy outcomes: miscarriage, *n* = 147; stillbirth, *n* = 34; premature birth, *n* = 2; neonatal death, *n* = 2; and jaundiced neonate, *n* = 13; among the pregnant women.

Various poor pregnancy outcomes such as miscarriage, stillbirth, premature birth, neonatal death, and icterus neonate were recorded among the pregnant women in their previous pregnancies. Overall, 19.8% of pregnant women reported poor pregnancy outcomes, with the highest outcome being miscarriage (74.2%). Majority of the poor pregnancy outcomes were recorded among the HEV seronegative pregnant women (Figure 3). Approximately 70.7, 6.6, 15.6, 1.0, and 1.0% seronegative pregnant women experienced miscarriages, neonatal jaundice, stillbirths, premature births, and neonatal deaths, respectively, in previous pregnancies. However, of the expecting mothers who tested positive for HEV IgG (8.3%), only 12% persons had poor pregnancy outcomes: approximately 8.4% had miscarriages and 3.6% had stillbirths.

The HEV IgG seroprevalence among the pregnant women in the Northern region was significantly higher than in all the other regions, whereas the seropositivity in the Eastern region was significantly greater than in the Volta Region. The seroprevalence in Accra, Volta Region and Central Region did not differ significantly from each other.

In logistic, both univariate and multivariate regression models, age, educational attainment, and region were significant predictors of HEV IgG seropositivity (Table 3).

**Table 3:** Regression analysis of demographic risk variables for HEV IgG infection in pregnant women.

Univariate logistic regression								
Variable	Coefficient	Standard error	*p*-value	OR	(95% CI)		Overall model fit	
					Low	High	$\chi^{2}$	*p*-value
Age	0.0594	0.0174	*0.0006*	1.0612	1.0259	1.0977	11.774	*0.0006*
Education	0.5418	0.0881	*<0.0001*	1.7192	1.4464	2.0434	39.742	*< 0.0001*
Employment							6.489	*0.1655*
Student	Ref	–	–	–	–	–		
Public Servants	1.73007	1.06242	*0.1034*	5.6410	0.7031	45.2591		
Traders	1.34515	1.02319	*0.1886*	3.8388	0.5167	28.5200		
Farmers	1.40464	1.06074	*0.1854*	4.0741	0.5095	32.5798		
Unemployed	0.85567	1.06343	*0.4210*	2.3529	0.2927	18.9156		
Region							105.502	*<0.0001*
Accra	Ref	-	-	-	-	-		
Eastern	0.56524	0.48651	*0.2453*	1.7599	0.6782	4.5668		
Central	−0.3467	0.59428	*0.5595*	0.7070	0.2206	2.2660		
Volta Region	−0.8678	0.69746	*0.2134*	0.4199	0.1070	1.6474		
Northern	2.37232	0.41574	*<0.0001*	10.722	4.7468	24.2198		
Multivariate logistic regression								
Age	0.07014	0.02202	*0.0014*	1.0727	1.0273	1.1200		
Education	0.31281	0.11063	*0.0047*	1.3673	1.1007	1.6983		
Employment								
Student	Ref	–	*–*	–	–	–		
Public servants	1.60699	1.11168	*0.1483*	4.9878	0.5644	44.0750		
Traders	2.09321	1.06076	*0.0485*	8.1109	1.0142	64.8640		
Farmers	0.31147	1.10505	*0.7781*	1.3654	0.1565	11.9100	151.017	*<0.0001*
Unemployed	1.94586	1.10422	*0.0780*	6.9997	0.8038	60.9547		
Region								
Accra	Ref	–	*–*	–	–	–		
Eastern	0.67367	0.49584	*0.1743*	1.9614	0.7422	5.1836		
Central	−0.1609	0.60228	*0.7893*	0.8513	0.2615	2.7718		
Volta Region	−0.7323	0.70412	*0.2983*	0.4808	0.1209	1.9111		
Northern	2.81040	0.45353	*<0.0001*	16.617	6.8310	40.4198		

Higher odds of HEV IgG seropositivity were associated with only the Northern Region (OR: 16.617; 95% CI: 6.8310–40.4198) compared with Accra in the multivariate model. 1.1 times higher odds (OR: 1.0727; 95% CI: 1.0273–1.1200) of HEV infection was associated with each increase in age from the lowest to the highest, while 1.4 times higher odds (OR: 1.3673; 95% CI: 1.1007–1.6983) was associated with education from the lowest to the highest in the multivariate model.

Among the behavioral risk factors, the source of drinking and the type of toilet used significantly predicted HEV IgG seroprevalence in both univariate and multivariate models, (Table 4). Higher odds (OR: 3.6843; 95% CI: 2.0508–6.6189) and (OR: 4.2281; 95%CI: 1.4989–11.9266) were associated with using pipe and borehole drinking water, respectively, compared with sachet water in the multivariate model. Also, there were higher odds of infection linked with using a public toilet (OR: 1.9921; 95% CI: 1.0717–3.7029) and open defaecation practice (OR: 2.3585; 95% CI: 1.3307–4.1801) compared with the use of home toilets. None of the obstetric factors such as the trimester of pregnancy (*p* = 0.2842), jaundice during pregnancy (*p* = 0.0.9982), jaundice in neonates at birth (*p* = 0.0.9976), and poor pregnancy outcomes including miscarriage, and stillbirth (*p* = 0.1387) showed a significant association with HEV seroprevalence in the univariate and multivariate regressions model except parity, *P* = <0.0001. HEV seropositivity increased with increasing parity with 1.4 odds (OR: 1.3565; 95% CI: 1.1592–1.5874) (Table 5).

**Table 4:** Regression analysis of behavioral risk variables for HEV IgG infection in pregnant women.

Univariate logistic regression								
Variable	Coeff	Standard error	*p*-value	OR	(95% CI)		Overall model fit	
					Low	High	$\chi^{2}$	*p*-value
Pork consumption							5.858	*0.0155*
No	Ref	–	–	–	–	–		
Yes	−0.6016	0.2577	*0.0196*	0.5479	0.3306	0.9080		
Veg consumption							1.278	*0.2582*
No	Ref	–	–	–	–	–		
Yes	0.6243	0.6030	*0.3005*	1.8670	0.5726	6.0879		
Drinking water							27.350	*< 0.0001*
Sachet	Ref	**–**	**–**	**–**	**–**	**–**		
Pipe	1.32863	0.29173	*<0.0001*	3.7759	2.1315	6.6887	–	–
Borehole	1.17784	0.50289	*0.0192*	3.2473	1.2119	8.7016	–	–
Rain	−16.771	6704.939	*0.9980*	0.0000	0.0000	0.0000	–	–
Well	0.05817	1.05196	*0.9559*	1.0599	0.1348	8.3313	–	–
Stream	0.40301	0.64645	*0.5330*	1.4963	0.4215	5.3125	–	–
Type of toilet							12.813	*0.0017*
Home	Ref	–	*–*	–	–	–		
Public	0.78471	0.30191	*0.0093*	2.1918	1.2128	3.9608	–	*–*
Open defaecation	0.89758	0.28132	*0.0014*	2.4536	1.4137	4.2587	–	*–*
Hand washing							0.065	*0.7986*
No	Ref	–	*–*	–	–	–		
Yes	−0.1036	0.4111	*0.8009*	0.9015	0.4028	2.0179		
Blood transfusion							0.293	*0.5881*
No	Ref	–	*–*	–	–	–		
Yes	−0.2313	0.4394	*0.5985*	0.7935	0.3354	1.8773		
Multivariate logistic regression								
Pork consumption								
No	Ref	–	–	–	–	–		
Yes	−0.7469	0.27407	*0.0064*	0.4738	0.2769	0.8108		
Veg consumption								
No	Ref	–	*–*	–	–	–		
Yes	0.82107	0.61910	*0.1848*	2.2729	0.6755	7.6485		
Drinking water								
Sachet	Ref	–	*–*	–	–	–		
Pipe	1.30407	0.29891	*<0.0001*	3.6843	2.0508	6.6189		
Borehole	1.44174	0.52910	*0.0064*	4.2281	1.4989	11.9266		
Rain	−16.606	6660.289	*0.9980*	0.0000	0.0000	0.0000		
Well	−0.0795	1.06063	*0.9402*	0.9236	0.1155	7.3840	48.716	*< 0.0001*
Stream	0.50640	0.65750	*0.4412*	1.6593	0.4574	6.0201		
Type of toilet								
Home	Ref	–	*–*	–	–	–		
Public	0.68921	0.31629	*0.0293*	1.9921	1.0717	3.7029		
Open defaecation	0.85802	0.29200	*0.0033*	2.3585	1.3307	4.1801		
Handwashing								
No	Ref	–	*–*	–	–	–		
Yes	−0.5592	0.42874	*0.1921*	0.5716	0.2467	1.3245		
Blood transfusion								
No	Ref	–	*–*	–	–	–		
Yes	−0.3984	0.45005	*0.3760*	0.6714	0.2779	1.6221		

**Table 5:** Regression analysis of obstetrics risk variables for HEV IgG infection in pregnant women.

Univariate logistic regression								
Variable	Coefficient	Standard error	*p*-value	OR	(95% CI)		Overall model fit	
					Low	High	$\chi^{2}$	*p*-value
Trimester	0.1692	0.15805	*0.2842*	1.1844	0.8689	1.6145	1.174	*0.2786*
Jaundice pregnancy							0.521	*0.4705*
No	Ref	–	*–*	–	–	–		
Yes	−17.608	7742.197	*0.9982*	0.0000	0.0000	0.0000		
Jaundice child							2.268	*0.1320*
No	Ref	–	*–*	–				
Yes	−18.619	6131.977	*0.9976*	0.0000	0.0000	0.0000		
Parity	0.3049	0.080184	*0.0001*	1.3565	1.1592	1.5874	14.60	*0.0001*
Poor outcome							2.453	*0.1173*
No	Ref	–	*–*	–	–			
Yes	−0.5148	0.34769	*0.1387*	0.5976	0.3023	1.1813		
Multivariate logistic regression								
Trimester	0.15124	0.16042	*0.3458*	1.1633	0.8494	1.5931		
Jaundice pregnancy								
No	Ref	–	*–*	–	–	–		
Yes	−19.322	21013.97	*0.9993*	0.0000	0.0000	0.0000		
Jaundice child								
No	Ref	–	*–*	–	–	–	22.23	*0.0005*
Yes	−19.682	10072.01	*0.9984*	0.0000	0.0000	0.0000		
Parity	0.3201	0.080410	*0.0001*	1.3773	1.1765	1.6124		
Poor outcome								
No	Ref	-	*-*	-	-	-		
Yes	−0.6227	0.35190	*0.0768*	0.5365	0.2692	1.0693		

Variables: Jaundice pregnancy = jaundice in mother during pregnancy; jaundice child = jaundice in child at birth; poor outcome = poor pregnancy outcome; parity = number of times given birth. Italicised values are *p*-values.

## Discussion

Increasing numbers of HEV seroprevalence studies have shown a high seroprevalence of infection among pregnant women. However, this study detected a much lower level of HEV infection in pregnant women in Ghana, with an overall HEV IgG seroprevalence of 8.3%. The HEV seroprevalence in pregnant women recorded in this study is among the lowest recorded in both Ghana and Africa so far.

The seroprevalence recorded in this study is lower than those reported by Obiri-Yeboah et al. (14) in Cape Coast (12.2%) and by Adjei et al. (13) in Accra (28.7%). The lower HEV IgG seroprevalence observed in this study compared to previous studies in Ghana may be attributed to the inclusion of pregnant women from various districts across different regions in Ghana, as well as a larger sample size. Thus, this larger study may constitute a more accurate estimate of HEV seroprevalence in pregnant women in Ghana than previous studies. Moreover, the use of the highly specific (22, 23) Wantai ELISA kit (Wantai Biological Pharmacy Enterprise Co., Beijing, China) may have given more reliable results than earlier test assays. Multiple studies have validated the Wantai assay, and it has proved to be of superior sensitivity than other commercial assays (23–25).

In this study, the HEV IgG seroprevalence of pregnant women in the Central Region of Ghana was found to be 2.5%, which is lower than the 12.2% reported by Obiri-Yeboah, Asante Awuku (14) in the same region. Also, the HEV IgG seroprevalence of 3.5% recorded in the Greater Accra Region in this study is lower compared with the HEV IgG seroprevalence of 18.5% recorded by Adjei et al. (13) in Accra. The seroprevalence in this study shows a wide range of within-country seroprevalence in pregnant women (1.5–28.0%). This wide within-country difference in seroprevalence among pregnant women in Ghana has also been reported in Egypt (45–84.3%), Ethiopia (31.1–58%), and Sudan (12.5–61.2%) (26).

In Africa, the HEV seroprevalence of 16.2–45% reported in Ethiopia, Nigeria, Eritrea, Egypt, Benin, and Sudan are also higher than that in this study (27–32). However, the HEV seroprevalence in this study is comparable to those reported in Burkina Faso (10.6%) (33), Cameroon (9%) (17), Tanzania (8%) (34), and Senegal 7.4% (35). The wide range of reported HEV seroprevalence values from these studies could be due to differences in hygiene practices, the endemicity of the virus in the study locations, and the level of exposure to risk factors for transmission over time. There are undoubtedly also differences in the sensitivity and specificity of assays used in previous studies. This indicates the clear need for a standardized WHO-certified assay for confirmation of HEV infection.

The HEV IgG seroprevalence is comparable to studies conducted in China (6%) (36) and Argentina (8.4%) (37).

The absence of HEV IgM and HEV RNA positives in this study, and the relatively low HEV antigen prevalence of 1.0% align with the low overall seroprevalence of 8.3% recorded in this study. This suggests a low burden of the disease in pregnant women. Given the fact that the sample size was calculated using HEV IgG seroprevalence data, this could potentially lead to an underestimation of IgM and RNA findings in cases of low positivity rates. We recommend that subsequent studies calculate sample sizes for each biomarker individually to improve statistical power.

However, the burden of HEV infection on pregnant women in the Northern region is significantly higher than the national average as revealed by the HEV IgG seroprevalence and HEV-Ag prevalence. The high HEV-Ag prevalence could be a result of frequent exposure to a source of HEV infection. Furthermore, there could be a low silent undetected outbreak in the region. In Ghana, sanitation conditions and the quality of drinking water supplies vary considerably across regions. The Northern Region is among the poorest in the country with correspondingly low development indices (38). It has the second-highest open defaecation rate in Ghana at 57% (39), which has a negative impact on WASH-related disease transmission. Such conditions may play a significant role in the variation in HEV seroprevalence among pregnant women in Ghana. Also, in situations of a small-scale, unrecognized outbreak of HEV in a community, high seroprevalence such as this is expected (40).

The finding of rising seroprevalence with age in this study is consistent with several studies of HEV seroprevalence in humans across the world (41–48). The observed trend of rising HEV IgG seroprevalence with age is well established to be the result of increased risk of exposure to risk factors of HEV and accumulated infections within a population over time (48–52). This is further emphasized by the half-life of HEV IgG. HEV IgG is a marker of exposure prevalence and tends to persist for extended periods (more than 10 years) (53).

Similarly, the association of increasing HEV seroprevalence with rising education level is influenced by age, as education level increases with increasing age. More of the younger women (<20 years) had basic school education while more older women (>40 years) were tertiary school graduates and illiterates.

Conversely, HEV seroprevalence rose with parity, which is correlated with increasing age. However, this finding may stem from the immunosuppressive effects induced by pregnancy over time due to successive pregnancies rather than age. Consequently, the greater the number of births a mother has, the higher the likelihood of experiencing HEV infections over time.

HEV is a disease associated with poor quality of drinking water and sanitation. Consistent with several reports around the world (30, 54–57), the toilet type, and the drinking water source were significant predictors of HEV IgG seroprevalence among the behavioral risk factors in the multivariate logistic regression model. The association of HEV seroprevalence with the use of unsanitary latrines has been reported by studies in Uganda (58) and Bangladesh (59). Open defaecation is a serious problem in many deprived communities in Ghana, with a rate of 57% in the Northern Region, where the majority of the seropositives were recorded. Of the majority of concern is the fact that open defaecation is sometimes practiced close to households, thereby creating avenues for easy contamination of food and water. The problem of open defaecation coupled with poor water, hygiene, and sanitation factors presumably are responsible for the high seroprevalence.

Several poor pregnancy outcomes that have been associated with HEV infection in pregnant women were recorded among the participants of this study. However, none was a significant predictor of HEV IgG seroprevalence. The fewer HEV seropositive pregnant women as compared with HEV seronegative pregnant women who had experienced these outcomes suggests that the outcomes are not the result of HEV infection. However, these poor pregnancy outcomes are unfortunately common among pregnant women in Ghana. These poor pregnancy outcomes certainly indicate that the health of pregnant women in Ghana needs to be improved and better antenatal care services provided for all women.

## Conclusion

HEV infections can have severe consequences during pregnancy. This study explored HEV seroprevalence and risk factors among pregnant women in Ghana. The HEV seroprevalence uncovered in pregnant women in this study is relatively low and similar to those reported in non-endemic countries. The low prevalence and seroprevalence of HEV, as well as the low level of HEV-related poor pregnancy outcomes among seropositive pregnant women, suggest a low burden of infection in pregnant women in Ghana. However, there is a high burden of infection in pregnant women in the Northern Region. While there is still a need for interventions to prevent HEV infection in pregnant women in Ghana generally, pregnancy care should be improved to achieve reduced maternofetal morbidity and mortality and expedite progress towards achieving goal 3 of the Sustainable Development Goals (SDGs).

Ghana currently does not routinely test pregnant women for HEV, and hepatitis B and C testing has limited implementation despite recommendations. However, donated blood is tested for hepatitis B and C, HIV, and Syphilis and is worthwhile in preventing accidental transfusion of infections. It is, therefore, important that policymakers within the health sector in Ghana include HEV in the standard national screening scheme for blood donors and extend it to cover pregnant women. Furthermore, the inclusion of HEV prevention and control within Ghana’s hepatitis eradication target, coupled with the promotion of public awareness regarding the threats associated with the disease, is imperative.

## Data Availability

The raw data supporting the conclusions of this article will be made available by the authors, without undue reservation.
